# Effects of Cardiac Rehabilitation on Cardiac Function and Cardiovascular Adverse Events in Coronary Heart Disease Patients Following Percutaneous Coronary Intervention: A Systematic Review and Meta-analysis of Randomized Controlled Trials

**DOI:** 10.31083/RCM39926

**Published:** 2025-09-26

**Authors:** Xingrong He, Jing Wang, Lingyan Ye, Linyan Xu, Jingquan Gao

**Affiliations:** ^1^School of Medicine, Lishui University, 323000 Lishui, Zhejiang, China; ^2^Department of Cardiology, The First Affiliated Hospital of Lishui University, Lishui People's Hospital, 323000 Lishui, Zhejiang, China

**Keywords:** cardiac rehabilitation, coronary heart disease, percutaneous coronary intervention, heart function, major adverse cardiovascular events

## Abstract

**Background:**

Assessment of the influence of the five central cardiac rehabilitation (CR) prescriptions on heart function and cardiovascular complications in individuals with coronary artery disease following percutaneous coronary intervention (PCI).

**Methods:**

This systematic review and meta-analysis was conducted and reported in accordance with Preferred Reporting Items for Systematic Reviews and Meta-Analyses (PRISMA) statement. A systematic search of PubMed, Web of Science, Ovid full-text journals database, Embase, CINAHL, Cochrane Library, MEDLINE, CNKI, VIP, SinoMed, and Wanfang data resources, was performed in November 2024. Studies that met the following criteria were included: (i) Population (P): adult individuals (18 years or older) with a confirmed diagnosis of ischemic heart disease using the clinical gold standard “coronary angiography” and undergoing first-time PCI; (ii) Intervention (I): five core cardiac rehabilitation prescriptions; (iii) Control (C): routine rehabilitation guidance/traditional rehabilitation guidance; (iv) Outcomes (O): left ventricular ejection fraction (LVEF)/left ventricular end-diastolic diameter (LVEDD)/six-minute walk distance (6MWD)/Major Adverse Cardiovascular Events (MACE); (v) Study design (S): randomized controlled trials.

**Results:**

This systematic review and meta-analysis of 16 randomized controlled trials (involving 1808 patients) demonstrates that comprehensive Cardiac Rehabilitation (CCR), integrating exercise training, nutritional counseling, smoking cessation support, psychological intervention, and medication management, yields two key benefits for coronary heart disease (CHD) patients following first-time PCI: (1) significant enhancement of cardiac function evidenced by improved LVEF (standardized mean difference (SMD) = 0.56, 95% confidence interval (CI): 0.33 to 0.79), reduced LVEDD (SMD = –0.67, 95% CI: –0.97 to –0.36), and increased exercise capacity (SMD = 0.82, 95% CI: 0.48 to 1.15); (2) substantial reduction in MACE (odds ratios (OR) = 0.15, 95% CI: 0.09 to 0.24).

**Conclusions:**

Patients who have undergone first-time PCI for CHD may experience significant advantages from the combined intervention of five CR strategies. Along with adherence to medical therapy and the evolution of medical models, strengthening multidisciplinary cooperation is crucial for optimizing clinical outcomes in patients following coronary interventional procedures.

**The PROSPERO Registration:**

CRD42024565139, URL: https://www.crd.york.ac.uk/prospero/display_record.php?ID=CRD42024565139.

## 1. Introduction

Coronary Heart Disease (CHD) is one of the most prevalent circulatory system diseases worldwide, with significant morbidity and mortality [1]. According to recent epidemiological data released by the World Health Organization (2021), cardiovascular conditions remain the leading cause of mortality globally, with annual fatalities reaching approximately 17.9 million, representing nearly one-third of total worldwide deaths [2]. It is projected that by 2030, deaths due to circulatory system diseases will exceed 23.6 million worldwide [3]. Statistics from the American Heart Association (2022) indicate that nearly 526 million individuals globally are affected by cardiovascular disease [4]. Despite a decline in global cardiovascular disease mortality rates from 1990 to 2019, diseases of the heart and blood vessels continue to be major cause of mortality, particularly affecting populations in less developed countries [5].

The economic impact of circulatory disorders in the U.S. affects both direct healthcare expenses and indirect societal costs, reaching an estimated annual total of $351.3 billion. By 2035, epidemiological studies suggest that 45.1% of the U.S. population could be affected by cardiovascular disease [6]. In China, total hospitalization costs for cardiovascular disease reached 165.222 billion yuan (1 US dollar ≈ 7.2476 Chinese yuan) in 2020, with the direct economic burden from Percutaneous Coronary Intervention (PCI) treatment for ischemic heart disease accounting for approximately 34.685 billion yuan. Since 2004, the growth rate of total hospitalization costs due to PCI has been 24.65% [7]. Data from the European Society of Cardiology (ESC) indicate that circulatory system diseases result in approximately 200 billion euros in direct and indirect costs in Europe [8]. In Japan, medical expenses for cardiovascular disease amounted to approximately 1.7 trillion yen in 2018 (1 US dollar ≈ 147.5400 yen), representing 11.5% of total medical expenditures [9]. A study projects that the worldwide economic burden of cardiovascular disease is expected to reach $883 billion by 2030 [10].

Although PCI serves as a primary treatment for CHD, first-time PCI recipients face unique challenges:pre-existing coronary artery stenoses or obstruction—resulting from pathophysiological changes induced by myocardial ischemia or necrosis—can significantly impair cardiac function. Furthermore, post-PCI complications, notably restenosis and persistent myocardial ischemia, may adversely impact the procedure’s long-term therapeutic efficacy. This population represents a clinically homogeneous cohort unaffected by prior revascularization—a methodological advantage that minimizes confounding from stent restenosis, graft failure, or chronic myocardial conditions seen in recurrent PCI patients [11].

Comprehensive Cardiac Rehabilitation (CCR), a multidisciplinary intervention strategy, addresses these challenges through its five core components: medication management, exercise prescription, nutritional counseling, psychological support (including sleep management), and patient education (focused on risk factor control and smoking cessation) [12]. Cardiovascular diseases are associated with a variety of risk factors, such as hypertension, hyperlipidemia, hyperglycemia, smoking, and psychological stress. Monotherapy (e.g., using only medication) can control certain risk factors but is not sufficient to comprehensively improve patients’ health conditions. The integrated use of the “Five Prescriptions” can target multiple risk factors simultaneously, thereby more effectively reducing the occurrence of cardiovascular events [13].

Cardiac rehabilitation (CR) is a well-established component of comprehensive CHD patient care, endorsed with Class I recommendations by major cardiology organizations including the Chinese Medical Association, American Heart Association (AHA), American College of Cardiology (ACC), European Society of Cardiology (ESC), and the National Institute for Health and Care Excellence (NICE) [14, 15, 16].

While RCT meta-analyses have demonstrated the efficacy of exercise-based CR in general PCI populations [17, 18, 19], the combined impact of all five CCR components specifically in first-time PCI recipients—a cohort where early intervention may yield maximal preventive benefits—remains unknown. This study therefore evaluates the comprehensive application of CCR on cardiac function and complications in this clinically distinct population.

## 2. Materials and Methods

### 2.1 Search Strategy

Computer research was used to study several databases, including PubMed, Web of Science, Ovid full-text journals database, Embase, CINAHL, Cochrane Library, MEDLINE, CNKI, VIP, SinoMed and Wanfang data resources, covering all records from their establishment through November 2024. The retrieval methodology employed a dual approach utilizing controlled vocabulary descriptors from the MeSH terms alongside free-text keywords. English search terms included “coronary heart disease/coronary disease/percutaneous coronary intervention/myocardial infarction/heart attack/myocardial infarct/cardiovascular stroke”, “cardiac rehabilitation/cardiovascular rehabilitation/cardiac rehabilitation activities/cardiac rehabilitation training/cardiac rehabilitation program”, “heart function/cardiac function/major adverse cardiovascular events”, “randomized controlled trial/clinical trials, randomized/trials, and randomized clinical”, Chinese search terms included corresponding terms for “coronary heart disease/PCI”, “cardiac rehabilitation”, “cardiac function/adverse cardiovascular events”, and “randomized controlled/effect/impact”, This study was designed and reported according to the Preferred Reporting Items for Systematic Reviews and Meta-Analyses (PRISMA) guidelines [20].

### 2.2 Study Selection

Studies that met the following criteria were included in this systematic review and meta-analysis: (1) Population (P): adult individuals (18 years or older) with a confirmed diagnosis of ischemic heart disease using the clinical gold standard “coronary angiography” and undergoing first-time PCI (to minimize heterogeneity from prior revascularization and ensure uniform baseline characteristics); (2) Intervention (I): five core CR prescriptions; (3) Control (C): routine rehabilitation guidance/traditional rehabilitation guidance (defined as: physician-guided outpatient visits without structured exercise prescription, consisting of (a) verbal medication adherence advice, (b) generic dietary recommendations, and (c) unsupervised walking suggestions); (4) Outcomes (O): left ventricular ejection fraction (LVEF)/left ventricular end-diastolic diameter (LVEDD)/six-minute walk distance (6MWD)/Major Adverse Cardiovascular Events (MACE); (5) Study design (S): randomized controlled trials. Exclusion criteria were: (1) unavailable full text or duplicate publications; (2) literature with unextractable statistical data; (3) non-Chinese or non-English literature.

### 2.3 Risk of Bias Assessment

Two independent investigators performed a rigorous quality appraisal of the selected studies utilizing the risk of bias evaluation instrument outlined in version 5.1.0 of the Cochrane Handbook for Systematic Reviews of Interventions [21]. The assessment framework encompassed six critical domains: ① randomization procedure implementation; ② concealment of allocation sequence; ③ masking of subjects, intervention providers, and outcome assessors; ④ description of loss to follow-up; ⑤ potential selective outcome reporting; ⑥ additional bias considerations. Disagreements between researchers were discussed until unanimous agreement was achieved. The methodological robustness of individual studies was categorized into three tiers according to bias potential: Grade A (low risk) — complete reporting of all specified criteria; Grade B (moderate risk) — partial fulfillment of reporting requirements; Grade C (high risk) — inadequate reporting of essential elements. When the evaluation results differed, a third researcher intervened for joint discussion, and finally only studies with quality grades A and B were included.

### 2.4 Data Extraction

Two researchers autonomously extracted data, and after discussion achieved unanimity to form the formal literature extraction table. Any discrepancies were adjudicated through consultation with an additional independent evaluator. Pertinent details were systematically recorded from each investigation, encompassing authorship details, publication year and geographic origin, demographic parameters (age and sex distribution), intervention measures, and outcome indicators.

### 2.5 Statistical Analysis

Statistical analysis was conducted utilizing Review Manager (RevMan, version 5.4; The Cochrane Collaboration, London, UK) and Stata (version 18.0; StataCorp LLC, College Station, TX, USA) Dichotomous outcomes were expressed as odds ratios (OR) or relative risks (RR), while for continuous data, mean difference (MD, or weighted mean difference, WMD) or standardized mean difference (SMD) were used as effect size indicators. Heterogeneity between studies was determined through the chi-square test, where *p* > 0.05 and I^2^ <50% suggesting acceptable homogeneity, warranting the application of fixed-effect models; In cases where *p* ≤ 0.05 and I^2^ ≥50%, indicating significant heterogeneity between subgroups, sensitivity analysis or subgroup analysis was conducted to identify potential sources of variability, or only qualitative analysis was performed. If heterogeneity could not be eliminated, random-effects models were implemented. A *p* < 0.05 indicated statistical significance. Potential publication bias was examined through funnel plot visualization of outcome measures across the included studies.

## 3. Results

### 3.1 Identified and Eligible Studies

**Fig. 1.**
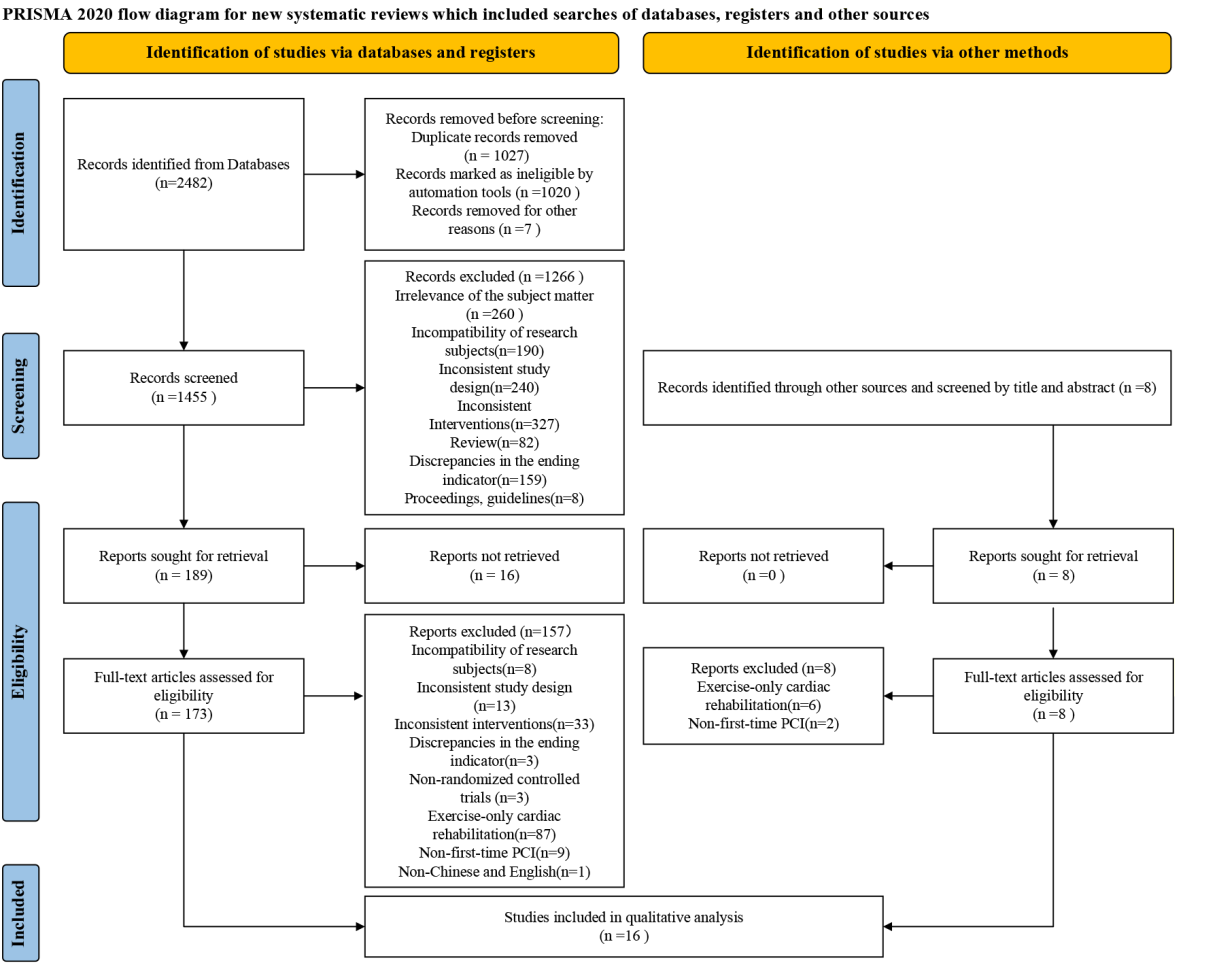
**PRISMA flow chart of study identification and selection**. PRISMA, Preferred Reporting Items for Systematic Reviews and Meta-Analyses; PCI, Percutaneous Coronary Intervention.

The initial search identified a total of 2482 articles. Using EndNote software (Version 21; Clarivate, Philadelphia, PA, USA), 1027 duplicate entries were eliminated. Following a thorough examination of titles and abstracts, an additional 1274 articles were deemed irrelevant and subsequently discarded. Upon conducting a comprehensive full-text assessment, 173 more articles were excluded. Ultimately, 16 studies fulfilled the selection criteria and were incorporated into the study (Fig. 1).

### 3.2 Characteristics of the Studies

The 16 investigations incorporated in the quantitative synthesis spanned publication years from 2017 to 2024, involving a total of 1808 patients. The geographical distribution of these studies included mainland China (n = 15) and the Islamic Republic of Iran (n = 1). All studies used CR as the intervention measure. The control groups received traditional rehabilitation measures (Table 1, Ref. [22, 23, 24, 25, 26, 27, 28, 29, 30, 31, 32, 33, 34, 35, 36, 37]).

**Table 1.** **Characteristics of studies included^1^**.

Auther, year	Country	Time	Group	Intervention	Population	Male/Female	Age (Years)	Outcome
Tian *et al*. 2020 [22]	China	3 M	Treatment	Home-based CR	53	32/21	63.49 ± 9.53	LVEF/6MWD
			Control	Usual care	53	30/23	63.0 ± 10.44	
Zhao *et al*. 2020 [23]	China	3 M	Treatment	CR	40	34/6	58.4 ± 10.1	LVEF/6MWD
			Control	Usual care	40	35/5	57.4 ± 9.4	
Xiong, 2021 [24]	China	1 Y	Treatment	CR	50	37/13	58.6 ± 5.8	LVEF/LVEDD/MACE
			Control	Usual care	50	32/18	61.7 ± 4.6	
Qian, 2023 [25]	China	3 M	Treatment	CR	100	68/32	65.80 ± 0.742	LVEF/LVEDD/MACE
			Control	Usual care	100	62/38	66.29 ± 0.689	
Du, 2023 [26]	China	6 M	Treatment	CR	45	26/19	62.71 ± 8.55	LVEF/6MWD
			Control	Usual care	45	27/18	64.04 ± 8.76	
Zhang *et al*. 2024 [27]	China	Not mentioned	Treatment	CR	60	37/23	60.57 ± 4.23	LVEF/LVEDD/6MWD
			Control	Usual care	60	35/25	60.36 ± 4.18	
Yang and Ge, 2021 [28]	China	Not mentioned	Treatment	CR	60	33/27	58.89 ± 9.70	LVEF
			Control	Usual care	60	35/25	58.63 ± 9.85	
Feng, 2020 [29]	China	1 W	Treatment	CR	40	28/12	58.4 ± 4.22	LVEF
			Control	Usual care	40	29/11	59.8 ± 4.47	
Yang and Liao, 2021 [30]	China	6 M	Treatment	CR	36	20/16	56.65 ± 3.40	LVEF/LVEDD/MACE
			Control	Usual care	36	22/14	56.77 ± 3.46	
Fang and Wang 2021 [31]	China	10–12 M	Treatment	CR	31	21/10	61.20 ± 11.33	MACE/LVEF/LVEDD
			Control	Usual care	31	23/8	57.33 ± 16.02	
Peng and Xu, 2021 [32]	China	12 M	Treatment	CR	53	29/24	57.60 ± 10.30	LVEF/LVEDD
			Control	Usual care	53	31/22	59.70 ± 9.60	
Lai *et al*. 2023 [33]	China	3 M	Treatment	Home-based CR	42	24/18	68.91 ± 6.37	MACE/LVEF/LVEDD/6MWD
			Control	Usual care	42	22/20	69.17 ± 6.45	
Zheng *et al*. 2024 [34]	China	3 M	Treatment	Home-based CR	53	32/21	63.49 ± 9.53	LVEF/6MWD
			Control	Usual care	53	30/23	63.0 ± 10.44	
Dorje *et al*. 2019 [35]	China	6 M	Treatment	Smartphone and social media-based CR	156	128/28	59.1 ± 9.4	6MWD
			Control	Usual care	156	126/30	61.9 ± 8.7	
Abtahi *et al*. 2017 [36]	Iran	10 W	Treatment	CR	25	14/11	53.76 ± 6.96	LVEF/LVEDD
			Control	Usual care	25	15/10	53.6 ± 6.98	
Zhong and Zhong J, 2022 [37]	China	3 M	Treatment	CR	60	35/25	45.86 ± 10.01	MACE/LVEF/LVEDD/6MWD
			Control	Usual care	60	38/22	45.96 ± 10.98	

Footnotes: ^1^ “Usual Care” in Chinese Studies (n = 15): (1) Post-PCI monitoring: 24-hour ECG surveillance, vital sign checks (BP/heart rate every 15–30 min initially); (2) Activity restriction: Bed rest for 24–48 hours, limb immobilization after sheath removal; (3) Discharge education: One-time session covering medication adherence (antiplatelets, statins), low-salt diet advice, and activity progression warnings; (4) Follow-up: Monthly outpatient cardiology visits without structured exercise prescription. “Usual Care” in Iranian Study (n = 1): (1) Risk factor management: Verbal instructions on smoking cessation, lipid control, and medication adherence; (2) No supervised exercise: Patients advised to “avoid strenuous activity” without specific exercise guidance; (3) Follow-up: Biweekly telephone consultations (10–15 min) focusing on symptom reporting. CR, cardiac rehabilitation; LVEF, left ventricular ejection fraction; 6MWD, six-minute walk distance; MACE, Major Adverse Cardiovascular Events; LVEDD, left ventricular end-diastolic diameter.

### 3.3 Risk of Bias Within Studies

**Fig. 2.**
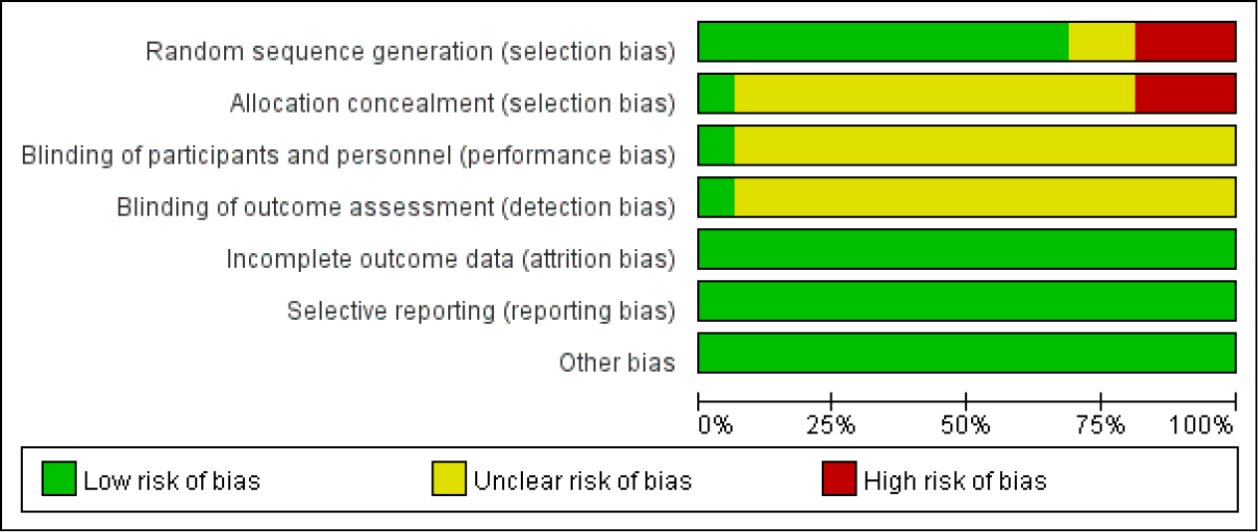
**Risk of bias assessment for studies included in the meta analysis**.

Among the 16 investigations incorporated in the quantitative synthesis, 1 study [30] used a lottery method, 2 studies [35, 36] used computer randomization, 3 studies [28, 31, 32] did not specify which randomization method was used, and 10 studies [22, 23, 24, 25, 26, 27, 29, 33, 34, 37] used a random number table method. 1 study [35] reported allocation concealment, while 3 studies [22, 26, 28] exhibited a high risk of bias in this domain. Given the difficulty in blinding CR interventions, 15 studies [22, 23, 24, 25, 26, 27, 28, 29, 30, 31, 32, 33, 34, 36, 37] did not conduct specific research on this aspect, while 1 study [35] had a low risk of bias in assessing participants, intervention providers, and outcome blinding. All incorporated studies demonstrated a low risk of bias in the reporting of outcome measures. 15 studies [22, 23, 24, 25, 26, 27, 28, 29, 30, 31, 32, 33, 34, 36, 37] were of evidence quality grade B, and 1 study [35] was of evidence quality grade A (Fig. 2).

### 3.4 Meta Analysis Outcomes

#### 3.4.1 LVEF

**Fig. 3.**
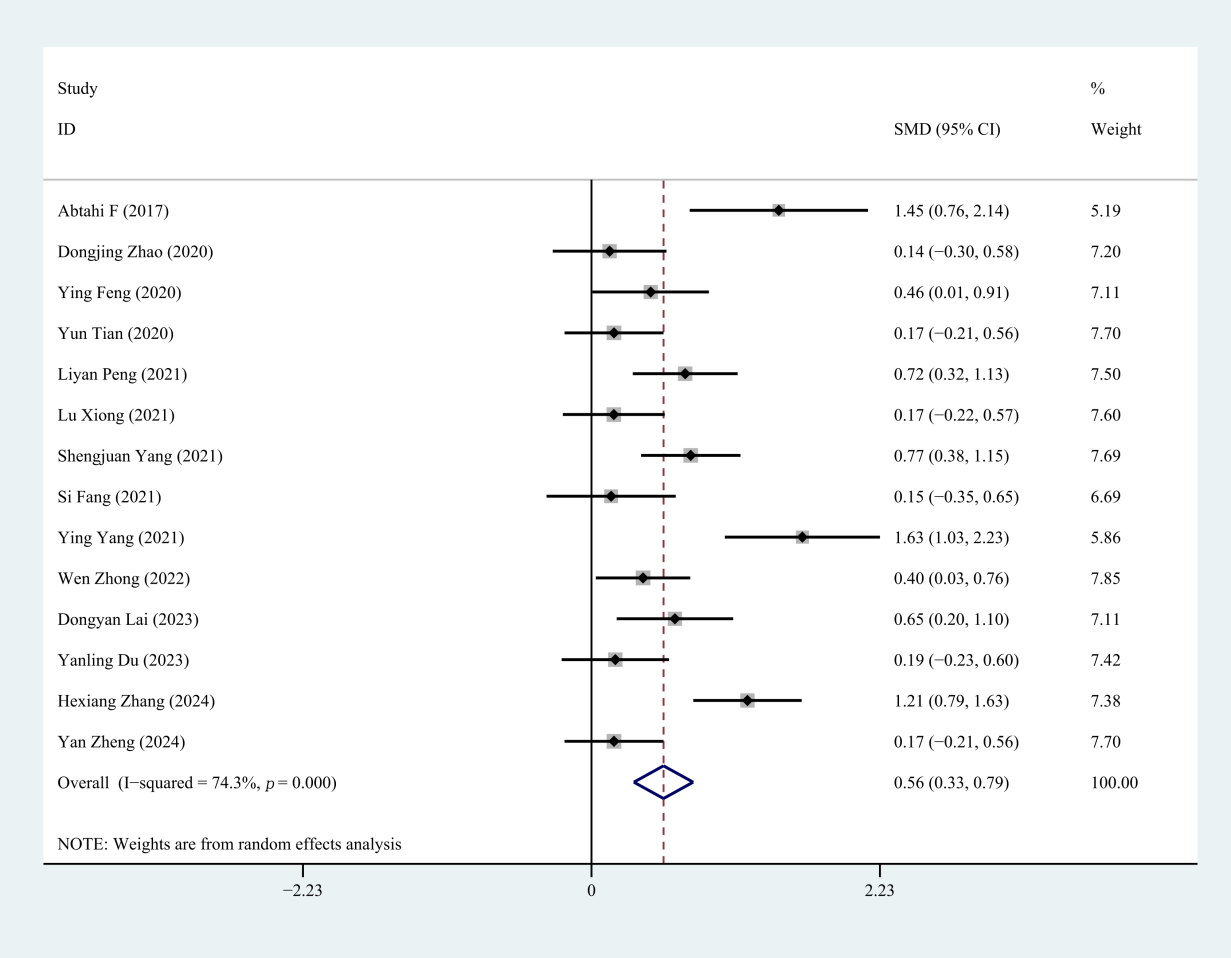
**Forest plot of LVEF**. LVEF, left ventricular ejection fraction; SMD, standardized mean difference; ID, identity; CI, confidence interval.

Fifteen studies reported LVEF status following CR. The heterogeneity assessment indicated significant variation across studies (*p* = 0.00, I^2^ = 93.7%). Sensitivity analysis demonstrated that removing the study by Qian [25] led to a marked reduction in heterogeneity, whereas excluding other studies had minimal impact on heterogeneity. After excluding Qian [25] , a random-effects model was applied. The heterogeneity test revealed persistent inter-study heterogeneity (*p* = 0.000, I^2^ = 74.3%). The results showed a statistically significant difference between the two groups (SMD = 0.56, 95% CI: 0.33 to 0.79, *p* = 0.000), suggesting that CR improved cardiac function in first-time PCI for CHD patients compared to the control group (Fig. 3).

#### 3.4.2 LVEDD

**Fig. 4.**
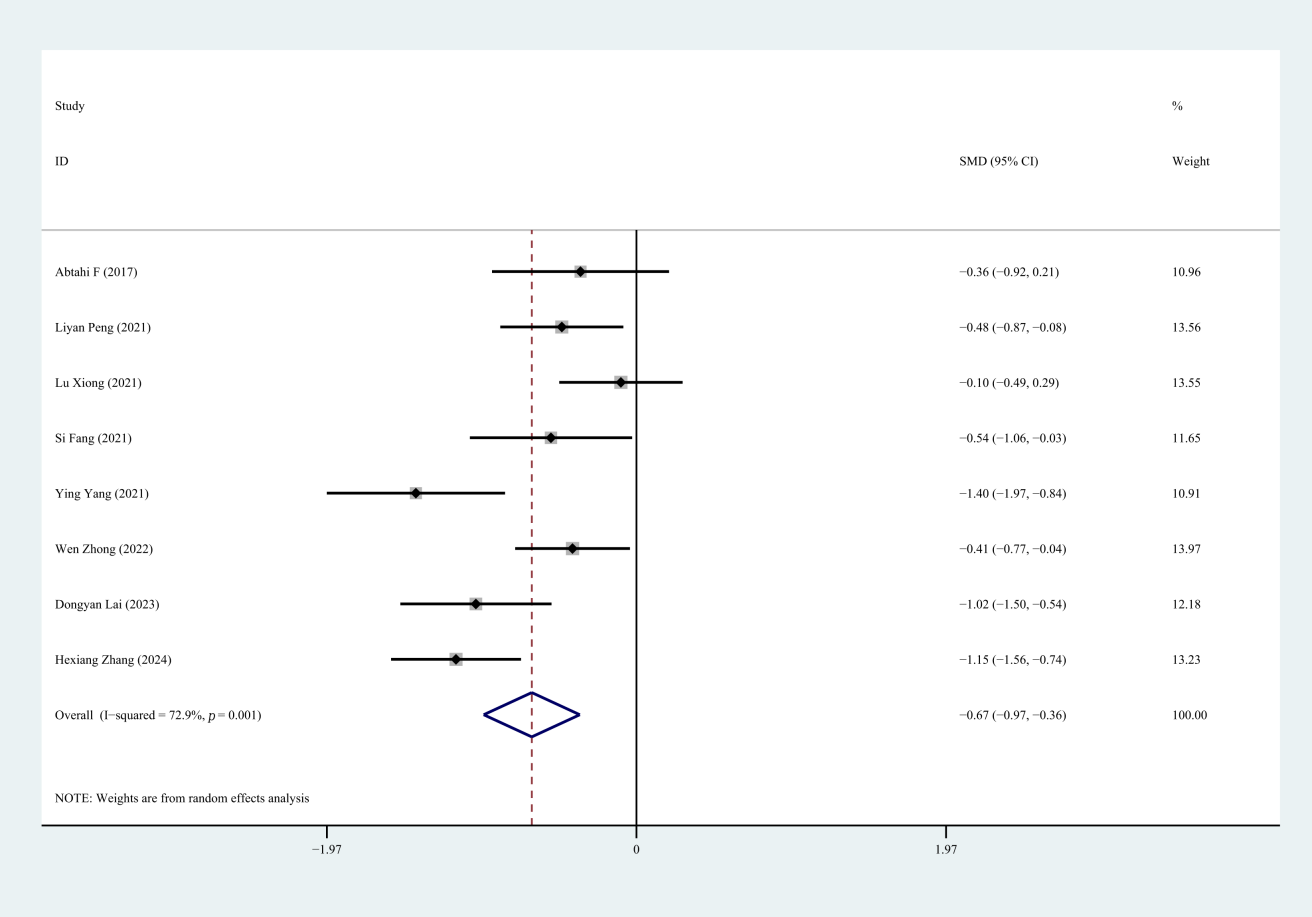
**Forest plot of LVEDD**. LVEDD, left ventricular end-diastolic diameter.

Nine studies reported LVEDD status following CR. Heterogeneity analysis revealed significant variation among the studies (*p* = 0.000, I^2^ = 95.4%). Sensitivity analysis demonstrated that removing the study by Qian [25] led to a substantial reduction in heterogeneity, whereas excluding other studies had minimal impact. After excluding Qian [25], a random-effects model was applied. The heterogeneity test indicated persistent inter-study heterogeneity (*p* = 0.001, I^2^ = 72.9%). The results showed a statistically significant difference between the two groups (SMD = –0.67, 95% CI: –0.97 to –0.36, *p* = 0.000), suggesting that CR can mitigate ventricular remodeling in first-time PCI for CHD (Fig. 4).

#### 3.4.3 6MWD

**Fig. 5.**
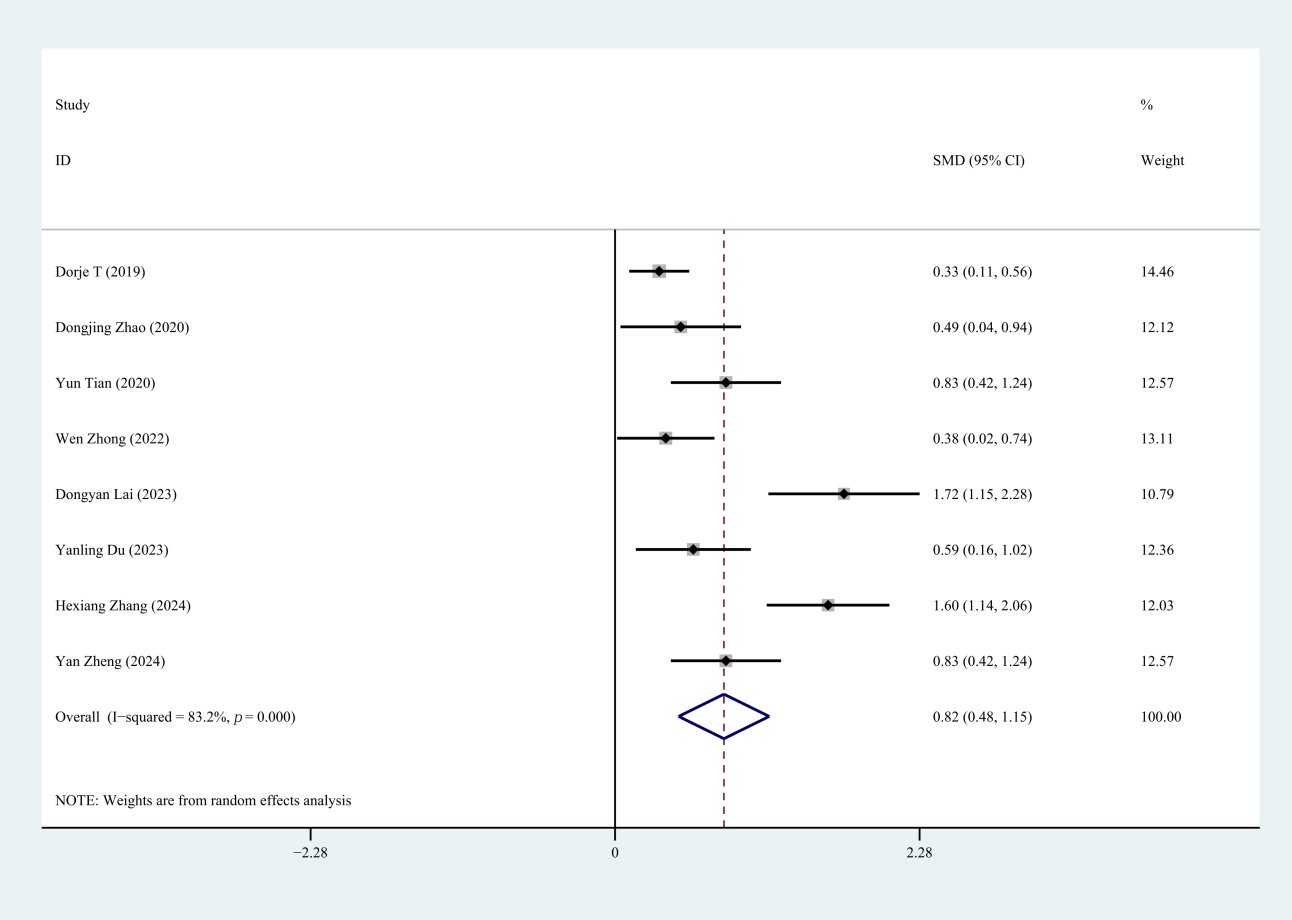
**Forest plot of 6MWD**. 6MWD, six-minute walk distance.

Eight studies reported 6MWD status after CR. Heterogeneity test analysis showed heterogeneity among studies (*p* = 0.000, I^2^ = 83.2%). Using random-effects model, the analysis showed statistically significant differences between groups (SMD = 0.82, 95% CI: 0.48 to 1.15, *p* = 0.000), indicating that CR can improve exercise tolerance in first-time PCI for CHD patients. Sensitivity analysis confirmed that removing any individual study would not affect the overall results, indicating the stability and reliability of the random-effects calculations (Fig. 5).

#### 3.4.4 MACE

**Fig. 6.**
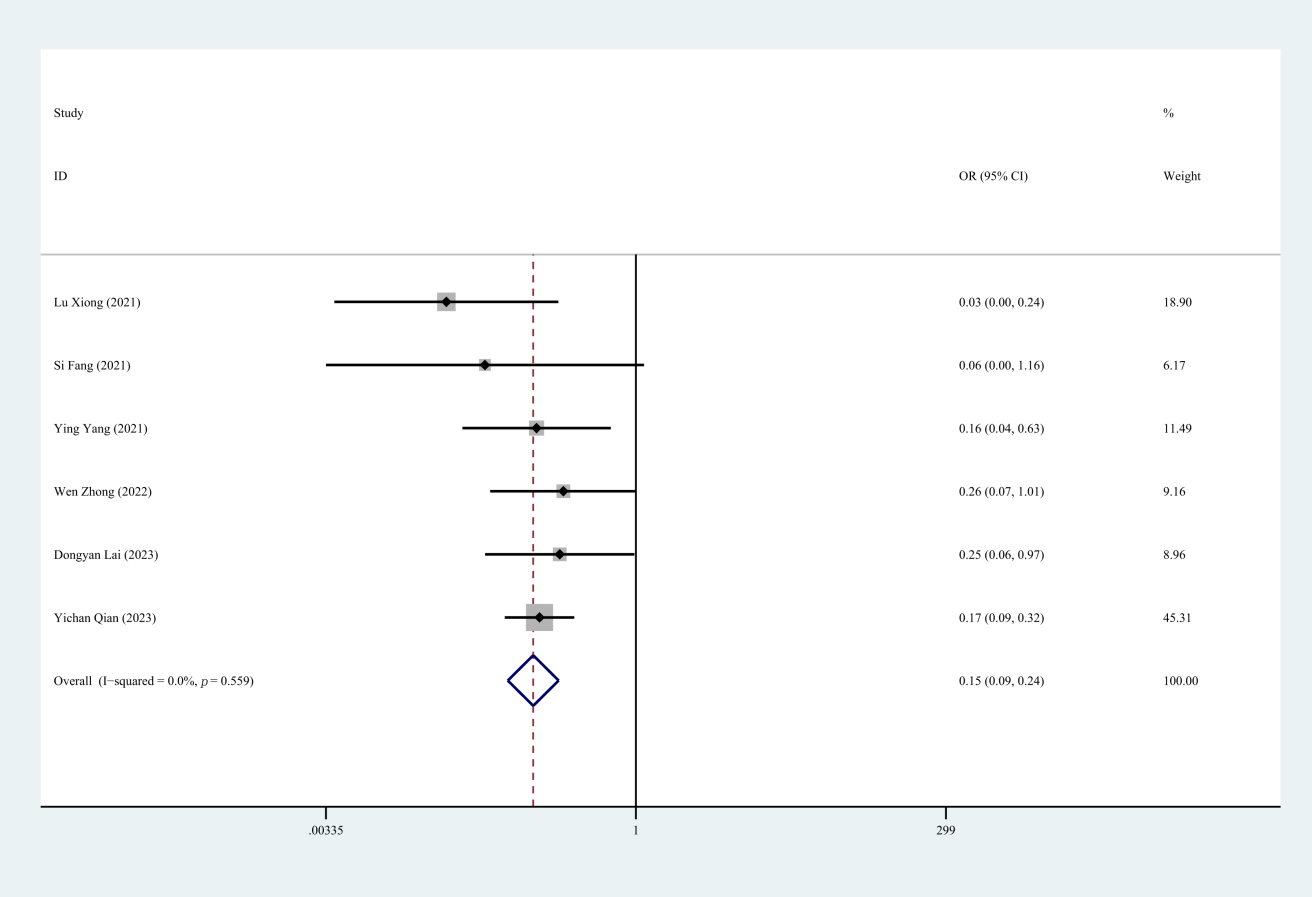
**Forest plot of MACE**. MACE, Major Adverse Cardiovascular Events; OR, odds ratios.

Six studies reported MACE during follow-up. The heterogeneity assessment indicated no significant variation across these studies (*p* = 0.559, I^2^ = 0.0%). The fixed-effects model analysis demonstrated a clinically significant between-group difference (OR = 0.15, 95% CI: 0.09 to 0.24, *p* = 0.000), indicating that CR interventions significantly reduce the occurrence of major adverse cardiovascular events in patients following first-time PCI (Fig. 6).

#### 3.4.5 Evaluation of Literature Heterogeneity

**Fig. 7.**
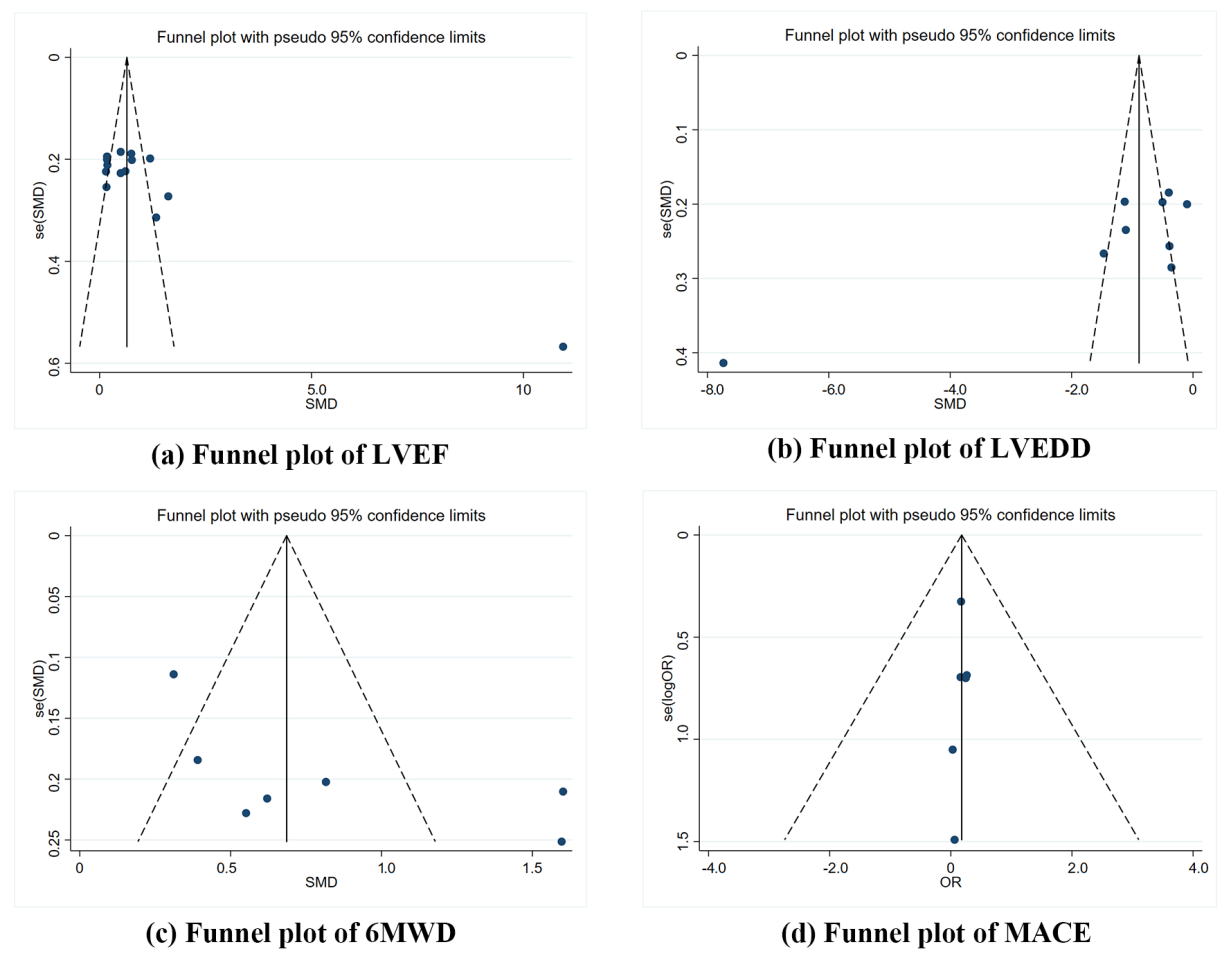
**Funnel plots**. (a) Funnel plot of LVEF. (b) Funnel plot of LVEDD. (c) Funnel plot of 6MWD. (d) Funnel plot of MACE.

The results indicated that the included literature had a slightly skewed overall distribution, suggesting possible publication bias. This may be related to unequal CR follow-up times, the significant heterogeneity in the intervention dosage and the treatment duration included in the study (Fig. 7).

## 4. Discussion

This systematic review and meta-analysis of 16 randomized controlled trials (involving 1808 patients) demonstrates that CCR, integrating exercise training, nutritional counseling, smoking cessation support, psychological intervention, and medication management, yields two key benefits for CHD patients following first-time PCI: (1) significant enhancement of cardiac function evidenced by improved LVEF (SMD = 0.56, 95% CI: 0.33 to 0.79), reduced LVEDD ( SMD = –0.67, 95% CI: –0.97 to –0.36), and increased exercise capacity (SMD = 0.82, 95% CI: 0.48 to 1.15); (2) substantial reduction in MACE (OR = 0.15, 95% CI: 0.09 to 0.24).

The results align with recent high-quality studies, such as the meta-analysis by Wen Zhong *et al*. [38], which demonstrated that home-based and technology-supported cardiac rehabilitation interventions significantly improve cardiovascular outcomes. Similarly, Antoniou *et al*. [39] highlighted the efficacy of structured rehabilitation programs in reducing MACE rates, further corroborating our findings.

Our findings are further supported by a recent meta-analysis by Zhang *et al*. [40], which specifically investigated the impact of CR initiation time and duration on post-PCI outcomes in AMI patients. Their analysis of 16 studies (including 1810 patients) found that neither the timing of CR initiation nor the program duration significantly affected improvements in LVEF, LVEDD, 6MWT, or the occurrence of cardiovascular events sunch as arrhythmias and angina. This aligns with our observation that variations in follow-up duration among included RCTs did not substantially influence the overall benefits of CR, reinforcing the significance of our conclusions regarding CR’s efficacy regardless of specific time parameters.

A Meta-analysis of RCTs [41] showed that the implementation of the five cardiac rehabilitation interventions led to significant improvements in myocardial function and a decline in the incidence of cardiovascular events among post-PCI coronary heart disease patients. Although smoking cessation and nutritional guidance were not included as part of the intervention measures in some studies, cardiac rehabilitation programs typically include reducing adverse lifestyle habits for improving treatment outcomes, such as reducing alcohol intake and encouraging smoking cessation, thereby reducing cardiovascular risks [42]. Furthermore, these trials demonstrated that better adherence to the five CR prescriptions can further reduce cardiac complication rates, as risk reductions of nearly 50% in cardiac events were not uncommon among the most compliant participants in some trials [43].

Attaining and sustaining a healthy body weight [44], adhering to nutritious dietary habits [45], engaging in consistent physical exercise [46], and quitting smoking [47] are each independently linked to a decreased risk of cardiovascular events.

The biological mechanisms underlying our results are well known. For example, improvements in LVEF and 6MWD can be attributed to enhanced myocardial efficiency and exercise capacity from consistent physical activity [48]. Similarly, reductions in MACE may stem from the combined effects of weight management, improved lipid profiles, and smoking cessation [49, 50, 51]. However, we acknowledge that variations in study designs, such as differences in exercise intensity may have influenced the observed risk reductions.

This study has some limitations: There is considerable heterogeneity in the intervention doses and treatment courses of treatment included in the study, and the funnel plot suggests the possibility of publication bias. Future studies should conduct multicenter, standardized randomized controlled trials with an extended follow-up period. Based on this study, clinicians can consider using CR therapy as an alternative option for PCI patients, however careful decisions should be based on individual circumstances.

## 5. Conclusions

This systematic review and meta-analysis revealed that combining five CR prescriptions—exercise, nutrition, psychological support, smoking cessation, and medication—significantly improves myocardial function and reduces cardiac complications in patients after PCI. Unlike previous studies focusing solely on exercise interventions, our findings emphasize the effectiveness of comprehensive rehabilitation approaches. The integration of these five prescriptions provides a personalized framework addressing the patients’ physiological, psychological, and social needs, highlighting the importance of multidisciplinary collaboration in CR. These findings suggest that implementing combined prescription strategies could optimize long-term outcomes for post-PCI patients. Future studies should focus on evaluating the efficacy of this integrated approach across various patient demographics to further refine rehabilitation strategies.

## Availability of Data and Materials

The datasets used and analyzed during the current study are available from the corresponding author on reasonable request.

## Supplementary Material

Supplementary material associated with this article can be found, in the online version, at https://doi.org/10.31083/RCM39926.
